# Successful delayed delivery of the second twin by evacuating the cord prolapsed first fetus and emergent cerclage: a report of 2 cases

**DOI:** 10.1186/s12884-022-04438-z

**Published:** 2022-02-10

**Authors:** Mia Park, Ye Won Jung, Jiwon Park, Soo Youn Song, Geon Woo Lee, Heon Jong Yoo, Young Bok Ko, Mina Lee, Byung Hun Kang

**Affiliations:** 1 Department of Obstetrics & Gynecology, Chungnam National University Hospital, Deajeon, Republic of Korea; 2 Department of Obstetrics & Gynecology, Chungnam National University Sejong Hospital, Sejong, Republic of Korea

**Keywords:** Twin pregnancy, Umbilical cord, Prolapse, Cervical cerclage, Premature rupture of membrane

## Abstract

**Background:**

In twin pregnancies, the cord prolapse of either fetus during the pre-viable period leads to fetal death but can also cause an intrauterine infection, leading to death or prematu-re birth of the remaining fetus. However, there are no validated protocols to prolong the gestational period or decrease the morbidity and mortality of the remaining fetus.

**Case presentation:**

The present cases were PPROM and cord prolapse very early during the second trimester (around 17 weeks in the first case and 19 weeks in the second case). The first fetus was evacuated, and cervical cerclage was performed at 23 and 20 weeks in the two cases, respectively. After maintaining the pregnancy, the second baby was born around 27 and 39 weeks in the first and second cases, respectively. The delivery interval between the first and second fetuses was 46 days in the first case and 126 days in the second case.

**Conclusion:**

If cord prolapse is identified at a pre-viable time in twin fetuses, evacuation and cerclage should be performed as soon as possible after the cord prolapse to reduce intrauterine infection and increase the survival chances of the remaining fetus.

## Background

The incidence of multiple gestation is increasing with the widespread use of assisted reproductive technology; this is accompanied by increased incidences of preterm birth, preterm premature rupture of the membranes (PPROM), and cord prolapse. Although very rare, umbilical cord prolapse can occur; it is a serious obstetric complication with reported incidence rates of 0.14–0.62% and perinatal mortality rates of 55–430 per 1000 cases [1]. Cord prolapse before 20 weeks of gestation in twin pregnancies can lead to death of the affected fetus, along with intrauterine infection that may result in death or preterm birth of the remaining fetus. Because umbilical cord prolapse is a potentially fatal obstetric emergency, prompt delivery (often by emergency caesarean section) is usually required for fetal survival; urgent pregnancy termination is necessary in cases that occur in the pre-viable fetal period because of the risks of chorioamnionitis and maternal sepsis. There have been several case reports of conservative management of cases that occurred in the pre-viable period, with the aim of extending pregnancy [2, 3]. In particular, such extension is optimal for mothers with twin pregnancy who have undergone assisted reproductive technology treatment, in whom a future pregnancy is not guaranteed.

Although there have been several case reports of extending pregnancy via conservative management of very early PPROM in twin pregnancy [4], there have been very few reports of healthy delivery of the remaining fetus by extension of the gestational period after cord prolapse of the affected fetus very early during the second trimester. There are two management approaches to this clinical condition: either the pregnancy period is extended through conservative management for delivery at the same time, or the pregnancy of the remaining fetus is extended after delivery of the affected fetus [5, 6]. Unfortunately, there is no validated protocol to prolong the gestational period or reduce the morbidity and mortality of the remaining fetus.

Here, we present two cases in which cord prolapse occurred in one fetus of dichorionic diamniotic twins during the pre-viable period. We successfully prolonged the pregnancy of the remaining fetus and delivered a healthy baby by evacuating the affected fetus and performing simultaneous cervical cerclage. We present these two cases along with a review of the relevant literature.

### Case presentaion

#### Case 1

A 36-year-old primigravida with dichorionic diamniotic twin pregnancy—confirmed during the first trimester, following conception by in vitro fertilisation-embryo transfer (IVF-ET)—was admitted at 16 weeks and 6 days with leaking liquor. Clinically, the leaking liquor was confirmed via speculum examination, but the cervical os was closed.

The mother’s initial vital signs were stable and she had no fever. There was no tenderness on the abdomen or uterus. The white blood cell (WBC) count was 11,600/µL and the C-reactive protein (CRP) level was 2.0 mg/dL. A high vaginal swab was taken, and the results were normal. Measurement of the amniotic fluid deepest vertical pocket showed that the first twin pocket had decreased. Broad-spectrum antibiotics were initiated to prolong the pregnancy; these comprised intravenous (IV) ceftriaxone at a dose of 2 g every 24 h, IV metronidazole at a dose of 500 mg every 8 h, and oral clarithromycin at a dose of 500 mg every 12 h. The WBC count (9,110/µL) and CRP level (0.4 mg/dL) were normalised after 2 days of antibiotic treatment.

**Fig. 1:**
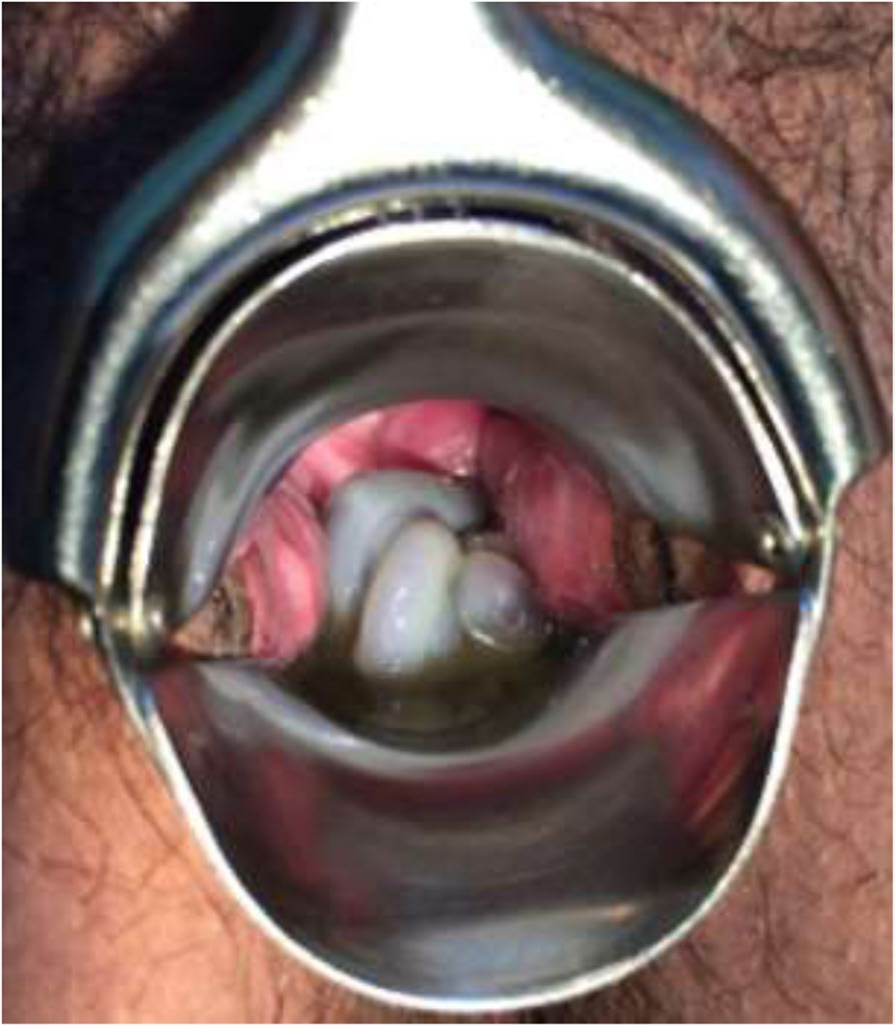
Photograph of the cord prolapse in the vagina

The first twin’s fetal cord was prolapsed at 17 + 2 weeks of gestation (Fig. 1). The patient complained that the umbilical cord protruded out of the vagina. Therefore, we performed speculum examination along with disinfection using sterile saline to prevent infection. We used the same antibiotic regimen, and there were no signs of infection or labour.

At 22 + 3 weeks of gestation (46 days after hospitalisation), delivery was imminent because of uterine contraction and cervical dilatation; thus, evacuation of the first fetus was performed. The first fetal placenta did not separate, so we decided to leave the placenta in the uterus. We began tocolytic administration of IV ritodrine, cut the umbilical cord of the first fetus as short as possible from the placenta, pushed the remaining umbilical cord into the uterus, and immediately performed cervical cerclage (McDonald method with two stitches). The delivered fetus died 1 day after admission to the neonatal intensive care unit (NICU). We continued tocolysis and broad-spectrum antibiotics while continuously monitoring the patient. No signs of infection were observed, and fetal monitoring was normal.

At 26 + 2 weeks of gestation (66 days after hospitalisation), which was 27 days after delivery of the first fetus, the second fetus was delivered by caesarean section because of uncontrollable preterm labour. The mother was discharged on day 5 after caesarean section without any sequelae.

Placental biopsy of the first baby showed acute gangrenous chorioamnionitis and funisitis, while placental biopsy of the second baby showed acute chorionitis. The surviving baby weighed 940 g and had Apgar scores of 7 and 9 at 1 and 5 min, respectively. The baby had mildly elevated inflammatory markers (WBC count 11,400/µL, CRP level 1.8 mg/dL, and procalcitonin level 1.45 mg/dL); therefore, it treated with prophylactic antibiotics. The baby had an uneventful neonatal course and was discharged on hospital day 85 weighing 2,960 g. The baby had bronchopulmonary dysplasia, but no oxygen therapy was needed after discharge. Regular follow-up as an outpatient revealed normal development without any other special complications at 5 years of age.

#### Case 2

A 34-year-old primigravida with dichorionic diamniotic twin pregnancy—confirmed during the first trimester, following conception by IVF-ET—was admitted to the obstetrics department at 18 weeks and 5 days because of leaking liquor. Her vital signs were stable; she had no symptoms or signs of infection, such as fever (WBC count 9,300/µL and CRP level 0.4 mg/dL). The fetuses were confirmed to be dichorionic diamniotic twins via ultrasonography, and the amniotic fluid of the first twin had decreased. Prophylactic broad-spectrum antibiotics were initiated.

**Fig. 2:**
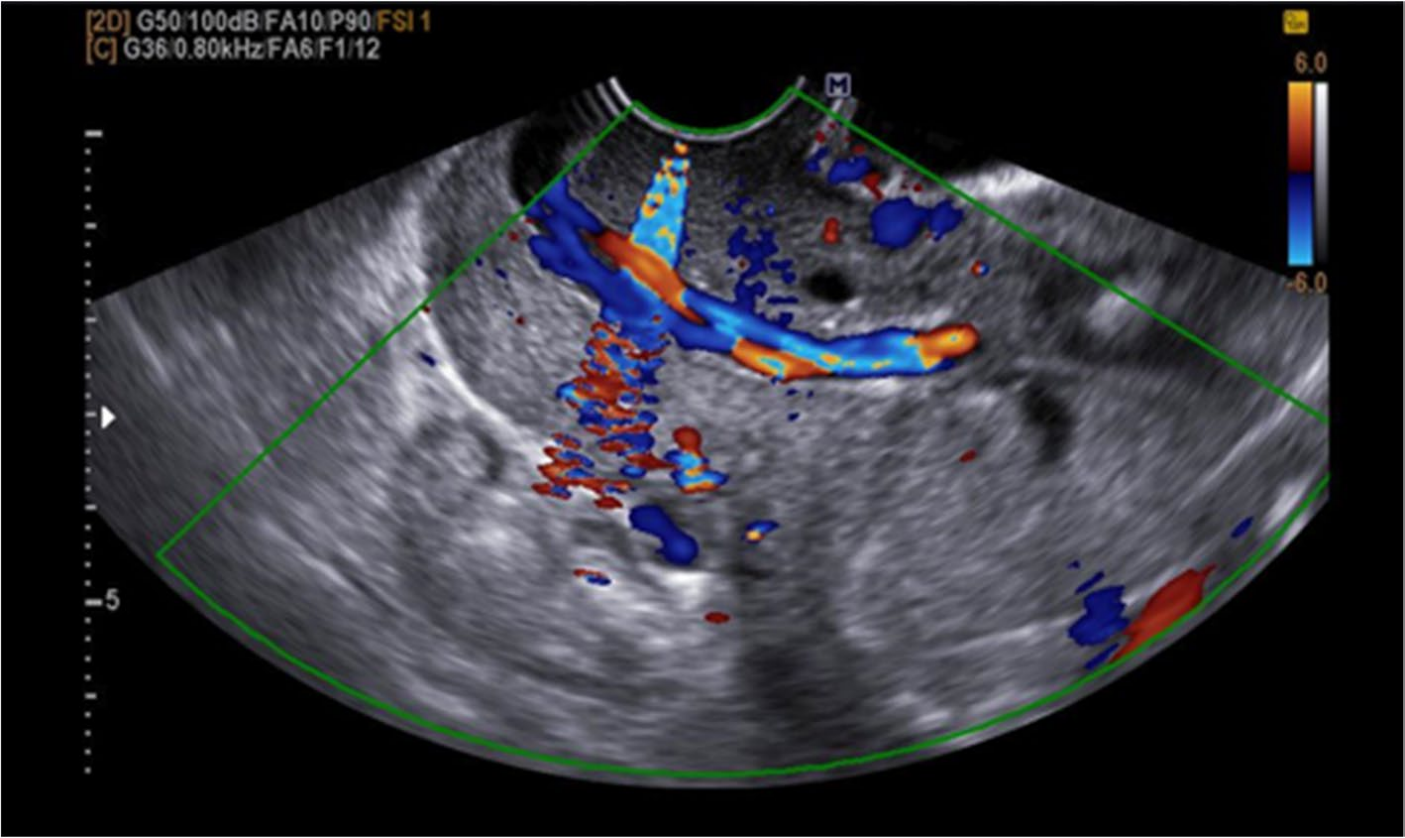
Color Doppler image demonstrating cord prolapse

At 19 + 6 weeks of gestation (9 days after hospitalisation), the umbilical cord of the first fetus prolapsed (Fig. 2), and the first fetus was found dead in the uterus on the next day. The mother had no uterine contractions; thus, we evacuated the dead fetus and retained the placenta in the uterus. We immediately performed cervical cerclage on the dilated cervix (McDonald method with two stitches). During the hospitalisation period, we continued the same broad-spectrum antibiotics as in Case 1. There were no signs of chorioamnionitis based on normal blood test results, clean vaginal secretions, no tenderness, and contraction-free uterus. The mother was discharged on day 17 after surgery, and no abnormalities were observed on subsequent outpatient monitoring.

**Fig. 3:**
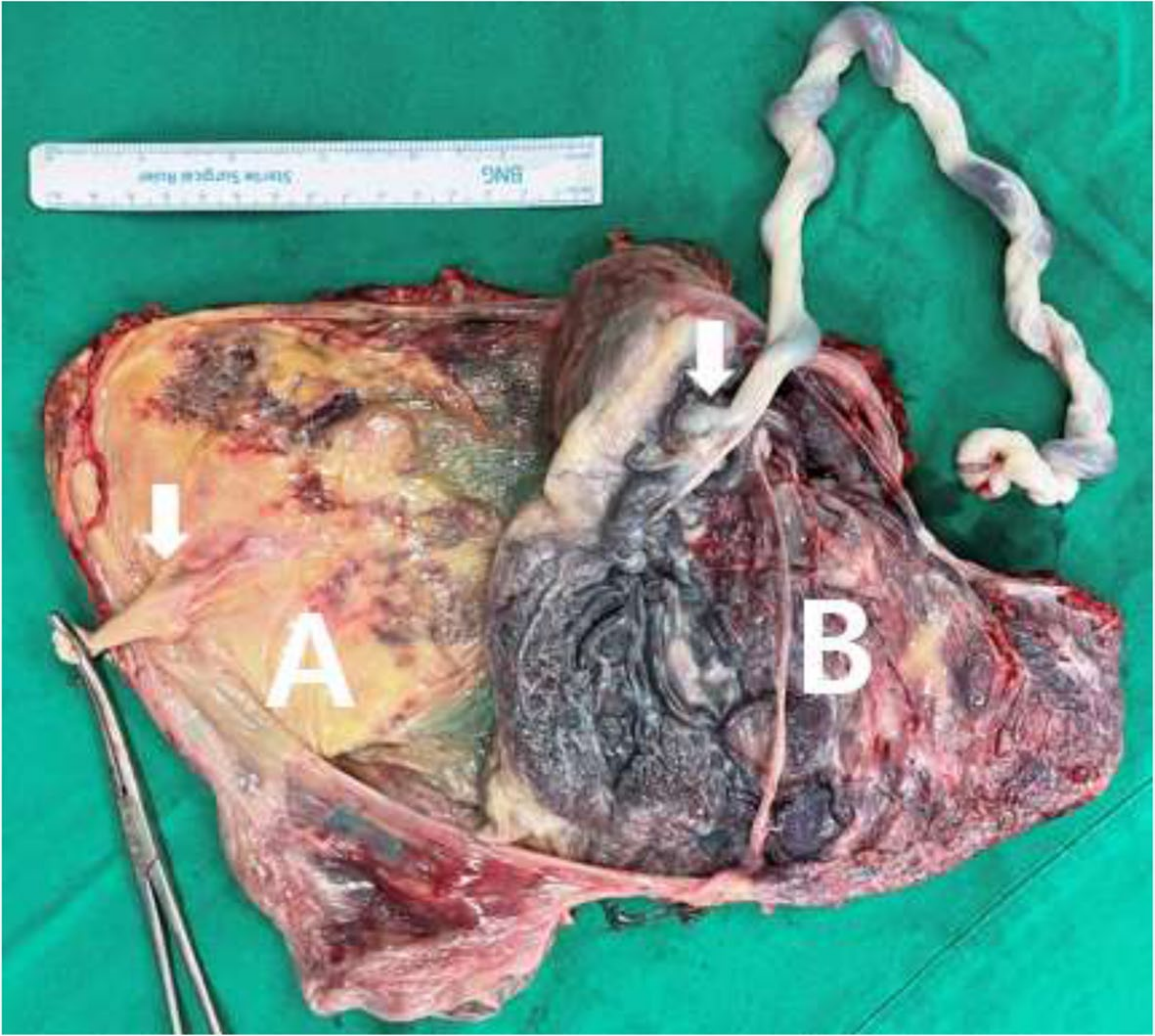
Gross post-delivery image of the dichorionic diamniotic placenta showing insertion of the umbilical cords (arrows). The remaining placenta of the first twin was atrophied (**A**), whereas the live-born second twin had a normal placenta (**B**)

The baby was delivered by elective caesarean section at 38 + 2 weeks of gestation. The baby weighed 3,220 g and had Apgar scores of 9 and 10 at 1 and 5 min, respectively. The first fetal placenta was atrophied; biopsy results showed that the parenchyma of the placenta was infarcted and hyalinised, while the second fetal placenta showed no signs of infection (Fig. 3). The baby is now 6 months old and healthy without any growth or developmental abnormalities. The mother is also healthy without any accompanying sequelae.

### Discussion and conclusions

The rate of twin pregnancies is increasing due to the wide use of artificial reproductive technologies, and perinatal complications are more frequent compared with singleton pregnancies. In the USA, the incidence of rupture of the membranes in twin pregnancies is 7.1% [7], of which 6.1% of cases involved a delayed second twin delivery at least 1 week after the first. The risk of cord prolapse is high in this situation. When the first twin’s cord prolapses, the babies are usually born together, and the pregnancy terminates very early during the second trimester. However, pre-viable gestation is a contraindication for immediate delivery in cases of umbilical cord prolapse [8]; in cases of twin pregnancy, despite the risk of infection and death of the affected fetus, it may be appropriate to delay the birth of the second twin when the next pregnancy is not guaranteed. Extending the pregnancy would be the best option for mothers with twin pregnancies who have undergone assisted reproductive technology treatment. However, this intervention is very rare [9]. The risk of expectant treatment is high if the pregnancy is maintained, which may lead to serious maternal or fetal infection and neonatal complications (e.g., endometritis, chorioamnionitis, and premature birth). Conservative treatment of umbilical cord prolapse should be reserved for carefully selected cases after the parents have received full counselling regarding the prognosis. Therefore, the clinical management of this very rare clinical condition varies among cases.

In a twin pregnancy, conservative management of PPROM early during the second trimester is very tricky. Pregnancies have been extended from 1 to 152 days, but more than 90% are delivered within 7 days [10]. The short latency period leads to extremely premature delivery; the neonate is at risk of morbidity/mortality-related preterm birth and infection [11]. In a previous report of conservative management, PPROM and cord prolapse of the first twin occurred at 24 weeks, while delivery of the second twin occurred at 28 weeks [9]. In another case, both fetuses were delivered at the same time at 28 weeks of gestation by extending the pregnancy period from 21 weeks after the first fetal cord prolapse [5].

One previous case report described surgical intervention. William et al. reported four cases of twin pregnancies complicated by PPROM and umbilical cord prolapse at 16–21 weeks of gestation [6]. They performed dilatation and evacuation of only the affected fetus under careful ultrasound guidance, to attempt delayed interval delivery; they achieved pregnancy extension by 42 to 133 days for the remaining fetus.

In our cases of PPROM, cord prolapse occurred very early during the second trimester at approximately 17 and 19 weeks in Cases 1 and 2, respectively. There was a longer latency to delivery of the second twin in both cases, with delivery intervals between the first and second fetuses of 46 days in Case 1 and 126 days in Case 2. The surviving baby in Case 1 was admitted to the NICU for 86 days, while the surviving baby in Case 2 was discharged from a regular neonatal unit (Table 1). The difference in neonatal outcome of the second twin may have been related to the interval between cord prolapse and evacuation/cerclage; evacuation and cerclage were performed at 36 days and 1 day after cord prolapse in Cases 1 and 2, respectively. Comparison of the two cases suggested that the interval from the first twin’s cord prolapse to delivery was related to the delivery time of the second twin.

**Table 1:** Characteristics of the remaining fetus after the first fetus was evacuated and cerclage performed in a complicated twin pregnancy

Case	GA at PPROM (weeks)	GA at Cord Prolapse (weeks)	GA at Cerclage (weeks)	GA at birth of the second baby (weeks)	Delivery interval (day)	Baby’s birth weight (g)	NICU admission (day)
1	16 + 6	17 + 2	22 + 3	26 + 2	46	940	86
2	18 + 5	19 + 6	20 + 2	38 + 2	126	3,220	0

*GA* gestational age, *PPROM* preterm premature rupture of membranes, *NICU* neonatal intensive care unit

Oyelese et al. studied the effects of delayed interval delivery on perinatal mortality; they reported that when the first twin was delivered at 22–23 weeks, delayed delivery of the second twin was associated with reduced perinatal and infant mortality of the second twin if the interval was less than 3 weeks [10]. In our cases, the delivery interval was more than 3 weeks in both cases through emergency cerclage; however, both second twins were relatively healthy.

Although the same antibiotics were administered in both cases, chorioamnionitis and funisitis were identified in the placental tissues in the first case, while no inflammation was apparent in the placental tissues in the second case. Preterm birth of the second twin was performed at a relatively early stage because of intrauterine infection; however, if no intrauterine infection is present, the pregnancy can be maintained to full term. There was no infection in the second twin’s placenta in either case.

Here, we presented very rare cases of the delivery of healthy neonates by extending the pregnancies via evacuation of the first twin and immediate cerclage, despite PPROM and cord prolapse occurring very early during the second trimester. These cases indicate that if cord prolapse is identified in the pre-viable fetal period in twin pregnancy, evacuation and cerclage should be performed as soon as possible to reduce intrauterine infection and increase the chance of survival for the remaining fetus.

## Data Availability

The datasets used and/or analysed during the current study available from the corresponding author on reasonable request.

## Abbreviations

PPROM: Preterm premature rupture of the membrane
IVF-ET: In vitro fertilization-embryo transfer
NICU: Neonatal intensive care unit
WBC: White blood cell
CRP: C-reactive protein
GA: Gestational age
IV: Intravenous
